# Isotope-specific lithium bioactivity – physiological reality or laboratory oddity?

**DOI:** 10.3389/fpsyt.2025.1664092

**Published:** 2025-09-15

**Authors:** Cecile Delacour, Marshall Deline, Hildigunnur Hermannsdóttir, Zhaoqi Lu, Michel J. P. Gingras, Tobias Fromme

**Affiliations:** ^1^ Institut Néel, Grenoble Alpes University, CNRS, Grenoble INP, Grenoble, France; ^2^ Chair of Molecular Nutritional Medicine, School of Life Science, Technical University of Munich, Freising, Germany; ^3^ Department of Physics and Astronomy, University of Waterloo, Waterloo, ON, Canada; ^4^ Waterloo Institute for Nanotechnology, University of Waterloo, Waterloo, ON, Canada

**Keywords:** lithium, lithium isotopes, bipolar disorder, amorphous calcium phosphate, quantum biology

## Abstract

The efficacy of lithium in treating bipolar disorder is well established, yet its precise molecular mechanisms remain elusive. A frequently overlooked dimension is the natural occurrence of two stable lithium isotopes (^6^Li and ^7^Li), which differ significantly in mass and nuclear spin and may therefore exhibit distinct bioactivity within living systems. Evidence from multiple rodent studies demonstrates isotope-dependent behaviour effects, suggesting translational relevance. Mechanistic exploration indicates that while classical lithium targets such as glycogen synthase kinase-3 beta and myo-inositol monophosphatase do not discriminate between isotopes, differential effects emerge at the level of mitochondrial calcium handling. Lithium isotopes modulate the calcium storage capacity of brain mitochondria, potentially via incorporation into amorphous calcium phosphate structures, which form crucial calcium depots within the mitochondrial matrix. The physical basis may involve isotope-dependent differences in mass or nuclear spin, possibly interacting with amorphous calcium phosphate or influencing radical pair formation, situating these findings within the rapidly expanding field of quantum biology. However, critical experimental gaps remain, particularly regarding whether isotope-specific mitochondrial effects translate to changes in neuronal signaling. Addressing these gaps through targeted physiological and clinical studies could clarify whether lithium isotope bioactivity is a laboratory curiosity or a tractable quantum biological phenomenon with therapeutic potential.

## Introduction

Lithium has been a cornerstone in the treatment of bipolar disorder and related mood disorders for decades (1). Despite its clinical efficacy, the precise molecular targets underlying its therapeutic effects remain under investigation. Classically, two main mechanisms are thought to be of importance: inhibition of glycogen synthase kinase-3 beta (GSK-3β) and modulation of myo-inositol monophosphatase. A third and more recent hypothesis focuses on mitochondrial function (2) and will be the primary focus of this review. Although lithium appears deceptively simple as a monovalent metal ion, its interaction with the various proposed molecular mechanisms remains rather incompletely understood.

A frequently disregarded aspect of lithium is the natural occurrence of its two stable isotopes, ^6^Li and ^7^Li, which might contribute differently to complex physico-chemical processes within living biomatter. The existence of multiple stable isotopes for a given element is not unusual and is observed in many biologically relevant elements. In the case of lithium, the lighter isotope (^6^Li), though less abundant, is still present at a substantial proportion (7.5% compared to 92.5% for ^7^Li). The mass difference between these isotopes is relatively large compared to isotope differences in heavier elements, and each differs in their nuclear spin (3/2 for ^7^Li versus 1 for ^6^Li). Each of these factors could plausibly result in differential bioactivity between the two isotopes – an aspect understandably overlooked in the clinical use of lithium salts, where the ^6^Li/^7^Li natural isotope ratio remains effectively constant.

Isotopes are generally assumed to behave nearly identically in a biochemical context, owing to their highly similar electronic structure. Indeed, isotopes are typically incorporated into biological systems without significant discrimination. Examples such as ^12^C/^13^C, ^14^N/^15^N, and ^16^O/^17^O/^18^O illustrate how isotopic variants are readily integrated into biomolecules and metabolic processes, with minimal selectivity (3). While isotopic selection does occur in biological and geological systems, its minuteness is consistent with the nearly identical electron configurations of isotopes of a given element and minor variations in atomic mass, which exert only subtle effects on bond lengths and strengths. Such small differences could account for slight isotope fractionation in living matter. This is not the case for the hydrogen (^1^H) – deuterium (^2^H) pair for which pronounced effects have been observed arising from the 100% mass difference that causes a large difference in zero-point quantum mechanical vibration energy, strongly affects the strength of chemical bonds, and leads to particularly strong fractionation (4). Isotopic effects can occur at the level of molecular interactions, where quantum effects are predominant. These isotopic effects can be attributed to differences in atomic mass affecting kinetic and thermodynamic properties or to differences in nuclear spin underlying effects of a nuclear nature. Interestingly, certain biological processes may rely on quantum effects, e.g. enzyme catalysis, photosynthesis, and olfactory sensing [reviewed in Ref (5)], and thus may be sensitive to isotopic effects of quantum mechanical nature.

The exact ratio of lithium isotopes has repeatedly been observed to deviate from the generally expected 7.5% ^6^Li to 92.5% ^7^Li ratio by a few per mile to several percent points in various systems, suggesting the presence of processes favoring one isotope over the other. These examples range from abiotic processes acting on marine basalts (6, 7), to more relevant examples of lithium isotope distribution within living organisms, *e.g*. in certain microalgae (8), absorption and excretion rates of cats (9), passage across the blood brain barrier in rats (10), and uptake by human erythrocytes (11). Furthermore, the different diffusion constants of lithium isotopes displayed *in vitro* (12) may be involved in differential *in vivo* compartmentalization.

## Evidence for differential lithium isotope effects on mammalian behaviour

Numerous studies have documented differential interactions of lithium isotopes with both abiotic and biotic systems. In this short review, we focus on neuronal-lithium interaction. Before exploring the mechanistic underpinnings that might cause such differences to manifest into mammalian behaviour, it is prudent to first ask whether there is evidence for the existence of any such effect in the first place. And indeed, multiple studies report different effects of ^6^Li versus ^7^Li on the behaviour of rats (13–16). In two studies in which lithium isotopes were delivered via the drinking water or injected, an entire battery of behavioural observables strongly differed between groups, including nest building, several parental care aspects, grooming, and alertness (14, 16). Aside from behavioural effects, ^6^Li also displays a higher toxicity in mice than ^7^Li (LD50 of ^6^Li: 13.2 mEq/kg; ^7^Li: 15.9 mEq/kg; natural Li: 14.9 mEq/kg) (20). While these differences seem small, they may be relevant due to the small therapeutic window of lithium concentrations in humans. This first qualitative evidence for isotopic behavioural effects gains clinical relevance through findings in a more translational rat model of mania – ketamine-induced hyperactivity – where similar differences in isotopic efficacy were also observed (15). Here, lithium was provided via highly palatable food, was ingested in a controlled amount, and led to similar measured plasma levels across isotopes. In measurements of spontaneous ambulatory activity, only the isotope ^6^Li was able to counteract ketamine-induced hyperlocomotion. Note that beyond new therapeutic possibilities, these behavioural studies provide the first demonstrations of isotopic lithium effect on mammalian behaviour.

## Interaction of lithium isotopes with plausible candidate mechanisms

Direct experimental investigation into the causes of lithium isotope-dependent effects on mammalian behaviour is complicated by the unresolved nature of mechanistic lithium targets. The mechanisms most commonly proposed as relevant have already been investigated using individual lithium isotopes.

Regarding the molecular scale, the most extensively studied target of lithium – yet still poorly understood – is GSK-3β (17, 18). However, neither activity nor phosphorylation state of this enzyme is differentially affected by lithium isotopes in a neuronal cell line (19). The same is true for another often assumed mechanism via myo-inositol monophosphatase, which is similarly inhibited by both ^6^Li and ^7^Li (20).

A further putative mechanism for lithium bioactivity has been proposed by Shalbuyeva and coworkers, namely an interference with mitochondrial calcium sequestration (21). In the presence of a high concentration of lithium, brain mitochondria displayed an altered capacity to transiently store calcium. This ability of mitochondria is central to synaptic calcium signaling and neurotransmitter release and thus neuronal communication underlying behavioural effects (22). Deline et al. tested the hypothesis of a divergent effect of ^6^Li and ^7^Li in isolated mitochondria of mouse brain and liver. In both types of mitochondria, lithium isotopes differently modified calcium storage capacity. In liver, lithium isotopes even differentially altered the susceptibility of mitochondria to the so-called *permeability transition*, a terminal phenomenon indicating calcium overload that was not observed in neuronal mitochondria under our experimental conditions (23). Interestingly, the direction of change was dependent on the tissue source (lithium decreased calcium storage capacity in liver and increased it in brain mitochondria), an observation in line with the well-known differing roles in calcium buffering between the high-capacity neuronal versus the low-capacity liver mitochondria (24). In both directions, ^7^Li invoked a greater change than ^6^Li compared to the potassium control. Changes to the calcium capacity of neuronal mitochondrial are thus the only known mechanistic component to date that may explain differential mammalian behaviour in response to ^6^Li versus ^7^Li.

## Unravelling the molecular clockwork

Many individual processes govern mitochondrial calcium buffering at the molecular level that might be sensitive to isotopic lithium effects. To better understand their complex molecular actions on intracellular signaling and rhythmic regulation, it is helpful to consider its potential influence across three interconnected levels: (i) the compartmentalization, such as how lithium distributes across cellular and subcellular spaces, (ii) the ion transport, and in particular its interactions with calcium and sodium ion channels and exchangers, and, finally, (iii) how it may affect mitochondrial calcium buffering and storage.

An obvious process is a differential compartmentalization across the mitochondrial inner membrane, where lithium may serve as a direct or indirect counter ion for calcium transport. As outlined above, there are multiple abiotic and biotic examples for a selective accumulation of one lithium isotope over the other. Most relevant here are studies that reported such effects across biomembranes (10, 11). Since none of these older reports specifically studied the structures relevant here, Deline et al. recently determined lithium isotope compartmentalization in mitochondria at rest and during calcium sequestration by state-of-the-art inductively coupled plasma mass spectrometry (ICP-MS) (23). Neither ion was selectively enriched in either case. The experiment was extended to mouse synaptosomes to study lithium isotope transport across the neuronal plasma membrane and, again, did not reveal any selectivity. The same remained true for lithium compartmentalization across living cells as determined by two-dimensional nanoscale secondary ion mass spectrometry (NANO-SIMS) and for cultured neurons. In short, none of the experimental models relevant to mitochondrial calcium sequestration displayed any isotope selectivity.

A possible lithium isotopic contribution could involve the sole known mitochondrial transporter exchanging lithium against calcium, the mitochondrial sodium/calcium/lithium exchanger (NCLX). In line with a lack of isotope fractionation, the NCLX proved ignorant of isotope identity (25). Also, the lithium compatible voltage-gated sodium channel did not discriminate isotopes in a patch clamp experiment (26). In summary, there is no evidence for direct or indirect isotope effects on membrane transport processes in the context of mitochondrial calcium sequestration, and, apparently, little room left to look for it.

What remains is the actual calcium depot within the mitochondrial matrix itself. Calcium is stored within the matrix in the form of gel-like amorphous calcium phosphate (ACP) to relieve concentration dependent import from excessive free calcium levels and to protect from hyperosmolar swelling (27–29). The building block of ACP is generally believed to be a highly symmetrical arrangement in the Ca_9_(PO_4_)_6_ stoichiometry, the so-called Posner cluster (30, 31). Interestingly, this cluster has been modelled to contain alternative cations, e.g. replacing the central calcium ion (32). Lithium turned out to be an energetically favorable substitution and may by that route influence both mitochondrial calcium, as observed earlier, explain isotopic differences (21, 23). Indeed, lithium integrates both into *in vitro* generated ACP and the presence of ^6^Li versus ^7^Li leads to differential formation of aggregate ACP (23, 33). The different properties of these ^6^Li-ACP or ^7^Li-ACP aggregates and their formation need to be further investigated to fully describe its role in different mitochondrial calcium storage capacity. These observations are a plausible root cause of isotopic lithium effects on mitochondrial ACP generation and storage capacity.

The physical reason for a different interaction of ^6^Li and ^7^Li with a surrounding Posner cluster is speculative. It may depend on the mass difference of the ions as well as on the differing nuclear spins. The latter has been proposed and theoretically modelled to form the basis of Posner cluster quantum characteristics (32, 34–37). Another theoretical framework proposes that the dissimilar nuclear spins of lithium isotopes might differently affect the formation of radical pairs (38). A dominant source of reactive oxygen species is the mitochondrial electron transport chain. A different effect of lithium isotope nuclear spin on radical pair formation within mitochondria may be an additional or alternative route towards altered mitochondrial calcium handling. For ease of reference, the evidence for the above isotopic effects and mechanisms is summarized in **Table 1**.

**Table 1:** Summary of evidence for lithium isotope-specific bioactivity.

Preferential distribution and uptake: Lithium isotopes are selectively taken up and distributed ion various tissues and bio;ogical fluids (plasama, cerebrospinal fluid, cerebral cortex, erythrocytes),often favoring ^6^Li	
9. Stokes et al. (19)	CSF/plasma ratio higher for ^6^Li, shorter half-life (12.9 h VS. 15.9 h)
10. Sherman et al. (10)	The cerebral cortex accumulates about 1.5 times more ^6^Li than ^7^Li, with no effect on inositol metabolism
11. Lieberman et al. (11)	Natural differences in isotopic abundance in erythrocytes suggest biological discrimination
12. Renshaw (12)	Measurable differences in diffusion constants in aqueous solution between ^6^Li and ^7^Li, contributing to isotopic effects
Distinct behavioural and toxic behaviors effects: ^6^Li often shows more pronounced therapeutic activity or toxicity, particularly in animal models of bipolar disorder-related	
13-14. Lieberman et al. (13)	Distinct biochemical and behavioural effects depending on the isotope (e.g., altered parental behaviour in rats)
15. Ettenberg et al. (15)	In a ketamine-induced hyperactivity model, ^6^Li more strongly and durably reduces hyperactivity than ^7^Li or natural lithium
16. Alexander et al. (16)	Lithium toxicity and behavioural effects vary according to isotope, ^6^Li being more toxic and more potent.
Neuronal modulation: Lithium isotopes similarly influence neuronal ion channels and potentially key enzymes (e.g., GSK-3β, myo-inositol monophosphatase)	
18. Livingstone (18)	In HT22 neuronal cells, no significant difference between ^6^Li and ^7^Li on GSK-3β activity or phosphorylation, nor on cell viability.
19. Livingstone et al. (19)	Confirmed in HT22 cells that ^6^Li and ^7^Li do not differ in toxicity, phosphorylation, or GSK-3β enzymatic activity
20. Parthasarathy et al. (20)	Similar effects of lithium isotopes on myo-inositol monophosphatase in multiple rat tissues.
26. Bukhteeva et al. (26)	Similar effects of natural lithium and its isotopes on voltage-gated sodium channel activity in SH-SY5Y neurons and iPSC-derived cortical neurons.
Mitochondrial mechanisms: ^6^Li and ^7^Li differentially modulate calcium dynamics, potentially impacting cellular function and signaling	
23. Deline et al. (23)	Lithium isotopes differentially alter the size distribution of amorphous calcium phosphate clusters in mitochondria, as well as mitochondrial calcium capacity, ^7^Li being the more potent isotope.
25. Bukhteeva et al. (25)	Similar isotopic effects on sodium/lithium co-transport and calcium efflux via the mitochondrial sodium/calcium/lithium exchanger.

If either mechanism were corroborated experimentally, lithium isotopic bioactivity may evolve to become a showcase of a highly relevant quantum biology phenomenon, a discipline that has rapidly advanced in recent years (5, 39, 40).

## A causal chain with missing links

**Figure 1:**
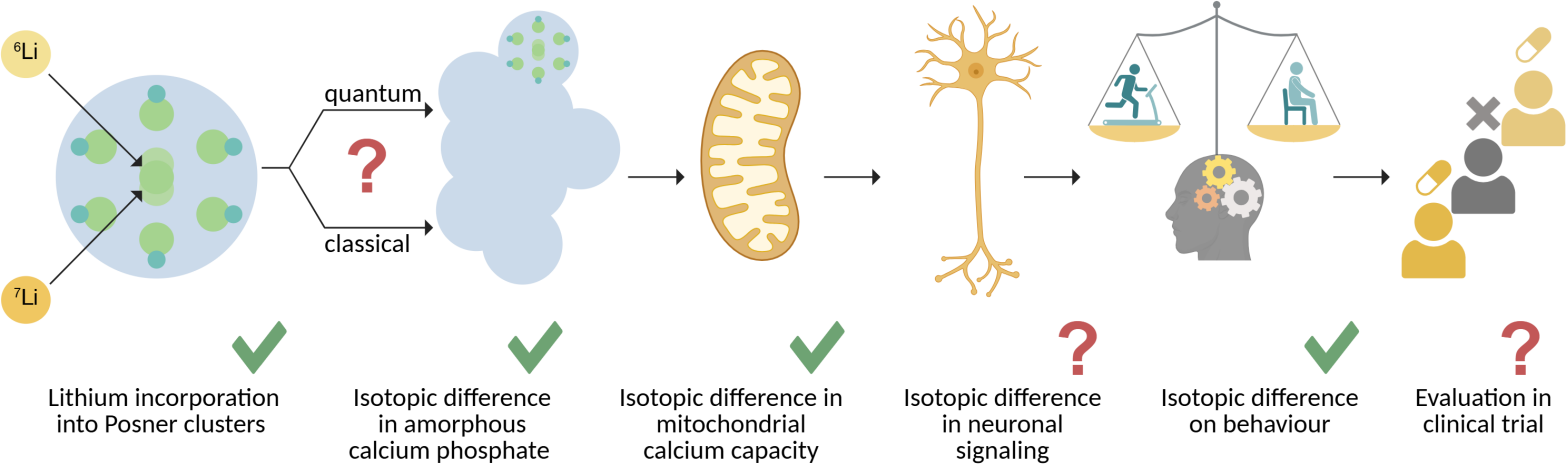
Overview on the mechanistic steps and gaps in the current understanding of lithium isotopic efficacy. Left to right: Lithium ions can enter the building blocks of amorphous calcium phosphate (ACP), the Posner cluster, and lead to differential aggregation by a yet unidentified – classical or quantum in nature – mechanism. This process plausibly underlies an established, differential isotope effect on the capacity of neuronal mitochondria to store calcium. It remains to be determined if a difference in mitochondrial calcium capacity manifests in altered neuronal signal processing, which in turn would provide a complete causal chain of events explaining lithium isotopic effects on mammalian behaviour. A clinical trial directly comparing lithium isotope therapeutic efficacy has yet to be conducted.

In the light of all of the above, a putative causal chain begins to take shape that mechanistically accounts for lithium isotopic effects on mammalian behaviour (**Figure 1**). At the smallest scale, lithium incorporates into the building blocks of ACP, via its key building block – the Posner cluster. Here, ^6^Li and ^7^Li cause a differential aggregation or functionality of the resulting amorphous calcium lithium phosphate, possibly mediated by their different spin and its interactions. This difference, in turn, manifests as a different calcium storage capacity and stability within the mitochondrial matrix.

It is plausible that this difference affects neuronal signaling due to the prominent role of mitochondrial calcium buffering in synaptic neurotransmitter release. While a large isotopic effect in a neurobiological context has recently been reported (44), it has yet to be experimentally tested in actual living neurons.

This caveat will have to be soon remedied, because it remains a key missing link within the causal chain of events linking molecular or even effective interactions of quantum mechanical origin with lithium isotopic bioactivity on mammalian behaviour. Finally, a dedicated clinical study objectively comparing the efficacy of each isotope in relevant medical indications remains to be performed. A deeper understanding of the process requires progress in both fundamental and clinical investigation.

## Conclusion

The initial question that inspired this article was whether the difference in lithium isotope bioactivity is a mere lab oddity or a reliable manifestation of quantum phenomena useful for clinical application. In the light of all collected evidence, we will for now have to settle on an unsatisfying “neither nor” or “to be determined”.

Regarding therapeutic applications, the question can ultimately be settled by the most authoritative tool: adequate clinical studies. The reasons why these have still not been performed are manyfold. One is that in an era of more and more complex pharmacological agents – biosimilars, RNA therapeutics, and many more – a simple metal ion inspires little investment enthusiasm. Another may be concerns of differential isotopic toxicity. A third is the current cost of pure lithium isotope salts which is orders of magnitude higher than that of natural lithium (approx. 250€/g ^6^Li or ^7^Li salts versus 1€/g natural (non-isotopically enriched) Li salts). Nevertheless, at an estimated 1 gram per day of intervention, the cost of such a hypothetical treatment would still be far less than the most expensive therapies on the market (41), especially considering that by its simple salt nature lithium requires virtually no prior cost intensive drug development. The supply situation may even further improve substantially in the coming decades, since enriched ^6^Li is envisioned to provide a substrate to breed tritium in all major concepts of current nuclear fusion research and eventually power generation (42, 43). A scaled-up worldwide lithium isotope separation capacity may in future provide more affordable amounts or either lithium isotope for clinical research and therapeutic application.

Can isotopic/quantum effects lead to systemic differences within a biological system? There undoubtedly are suggestive pieces of evidence in place – however, these do not yet form a complete mechanistic chain of causal events. In a bottom-up perspective, lithium isotopes differentially interact with amorphous calcium phosphate that in turn plausibly underlies an isotope specific mitochondrial calcium capacity. It remains unresolved if these changes are of sufficient magnitude to affect neuronal signal transduction in general or at least in specific neurophysiological situations. Such experiments are vitally called for to causally connect lithium isotope specific bioactivity on the abiotic and organellar level with the observed difference on the level of mammalian behaviour.
